# Caregiver-reported availability of home learning materials for toddlers in underserved communities

**DOI:** 10.3389/fpubh.2026.1899274

**Published:** 2026-08-19

**Authors:** May Oo, Shelby Anderson-Badbade, Peyton Rogers, Rebecca Emery Tavernier

**Affiliations:** 1 Moses/Weitzman Health System, Inc, Middletown, CT, United States; 2 University of Minnesota Twin Cities School of Medicine Duluth Campus, Duluth, MN, United States

**Keywords:** availability of learning materials, federally qualified health centers, home environment, toddlers, underserved communities

## Abstract

**Objective:**

To describe caregiver-reported availability of toddler-appropriate learning materials among underserved families and examine short-term changes in reported availability.

**Background:**

The home learning environment includes structural resources, such as toys, books, and learning materials, that may support early childhood development. Although prior research has examined overall cognitive stimulation and caregiver-child interactions, less is known about specific types of learning materials and how their availability changes over time, particularly among families receiving care in safety-net settings.

**Methods:**

This secondary analysis included 148 caregivers of 18–36-month-old children recruited from two Federally Qualified Health Centers in Florida and New York as part of a larger pilot study of the Prescription for Play (P4P) program. Caregivers who did (*n* = 38) and did not (*n* = 110) receive P4P completed the Availability of Learning Materials (ALM) subscale of the StimQ_2_ Toddler at baseline and three-month follow-up. The ALM assesses the presence of learning materials across five domains: Symbolic Play, Art, Adaptive/Fine Motor, Language, and Life-Size Play. Because P4P was not designed to modify home learning materials and no intervention effects were detected, caregivers were analyzed as a single cohort regardless of P4P exposure. Paired-samples *t*-tests examined within-family changes over time.

**Results:**

Caregivers reported relatively high availability of learning materials across all ALM domains at both time points. Significant increases were observed in total ALM scores (*p* < 0.001, Cohen’s *d* = 0.44), as well as in Symbolic Play (*p* < 0.001, Cohen’s *d* = 0.49) and Art (*p* = 0.007, Cohen’s *d* = 0.28) domains. Changes in the remaining ALM domains were small and did not reach statistical significance (*p*s > 0.05, Cohen’s *d*s < 0.16).

**Conclusion:**

Caregivers from safety-net pediatric settings reported the availability of a broad range of toddler-appropriate learning materials, with modest increases over a 3-month period. These findings suggest that caregivers provide their toddlers with a range of developmentally supportive learning materials. However, future research is needed to clarify whether the availability of such materials translates to enhanced material use, caregiver-child interaction quality, or child developmental outcomes.

## Introduction

The home learning environment plays a central role in early childhood development by shaping children’s opportunities for exploration, play, and learning. It is a multidimensional construct that includes both structural resources, such as books, toys, and learning materials, and interactive processes, such as shared reading, caregiver verbal responsiveness, and play engagement, that together contribute to children’s cognitive stimulation and development (1, 2). Although these components are related, they represent distinct aspects of children’s developmental experiences. Structural resources provide opportunities for learning and exploration, whereas interactive processes reflect how caregivers and children engage with those resources (2, 3). Understanding the availability of structural resources is therefore important for characterizing children’s home learning environments and identifying opportunities to support early development.

Structural resources within the home learning environment have consistently been associated with children’s cognitive, language, and socioemotional development (1, 4, 5). Despite their importance, these resources have often been assessed as part of broader composite measures of cognitive stimulation, making it difficult to isolate the contribution of specific types of learning materials (6). In addition, although observational assessments provide detailed information about home learning resources, they are time- and resource-intensive, limiting their feasibility for large-scale research and routine clinical settings (7). Brief caregiver-report measures that assess specific domains of structural resources therefore provide a practical approach for characterizing home learning materials across diverse populations.

One such brief measure is the Availability of Learning Materials (ALM) subscale of the StimQ, which assesses the presence of developmentally appropriate materials across five domains of play and learning: Symbolic Play, Art, Adaptive/Fine Motor, Language, and Life-Size Play (3, 8). The StimQ was originally developed and validated among families from low-income urban communities to assess cognitive stimulation within the home environment (9). Subsequent versions, including the Infant StimQ and Toddler StimQ/StimQ_2_, have been widely used in pediatric and early childhood research, particularly among populations experiencing socioeconomic disadvantage (3, 8). Validation research demonstrates that higher ALM scores are associated with children’s cognitive ability, expressive and receptive language, and social–emotional competence, including among predominantly low-income families (3, 10). Additionally, caregiver characteristics, including maternal literacy and education, have been associated with differences in the availability of learning materials (30). Although the ALM does not directly assess caregiver-child interactions or child development, its associations with developmental outcomes support its use as an indicator of material-based cognitive stimulation within the home learning environment (3).

Despite extensive use of the StimQ to assess the home learning environment, several important gaps remain in understanding the availability of home learning materials. First, previous studies based in pediatric primary care have often focused on overall ALM scores rather than on specific domains of learning material availability. For example, studies of the Video Interaction Project used the ALM subscale to assess the availability of learning materials in the home and reported overall ALM scores as an outcome, but did not characterize the availability of specific categories of learning materials (e.g., Symbolic Play, Adaptive/Fine Motor, Art, Language, and Life-Size Play) (11, 12). Similarly, a hospital-based study using Infant, Toddler, and Preschool StimQ measures characterized home environments across developmental stages and primarily reported overall StimQ scores rather than detailed ALM domain-specific distributions (13). Consequently, less is known about the availability of specific domains of learning materials among families receiving pediatric care.

Second, longitudinal research has shown that the home learning environment changes across early childhood (14), yet relatively little is known about short-term, within-family changes in caregiver-reported availability of learning materials or changes in specific ALM domains. Toddlerhood represents a particular period of rapid developmental change, during which children acquire new cognitive, language, motor, and social skills and their play interests evolve (15, 31). As children’s developmental abilities change, caregivers may introduce, replace, or expand learning materials available in the home. Although longitudinal research suggests that aspects of the home learning environment change across early childhood (14), less is known about whether specific structural components of the home learning environment demonstrate measurable changes over relatively short periods within families. Characterizing these change may provide insight into the stability and evolution of structural learning resources available to young children.

These gaps are particularly relevant among families receiving care in safety-net healthcare settings, who have rarely been targeted in prior research using the ALM. Safety-net settings provide healthcare services to populations who may experience barriers related to income, insurance coverage, or access to healthcare and social resources. Federally Qualified Health Centers (FQHCs) are a core component of the pediatric safety net, serving approximately 1 in 8 U.S. children (16) and providing preventive healthcare, developmental surveillance, and anticipatory guidance for early childhood development (17, 18). Families receiving care in these settings may face socioeconomic barriers that limit access to structural resources; however, socioeconomic disadvantage does not necessarily imply the absence of supportive caregiving practices or structural resources (10). Strengths-based perspectives in developmental science emphasize identifying existing family assets, adaptive caregiving practices, and community resources while acknowledging structural inequities that shape children’s developmental opportunities (19–21). Applying this perspective to the home learning environment highlights the importance of characterizing both existing developmental resources and areas where additional support may be beneficial.

This secondary analysis addresses these gaps by characterizing caregiver-reported availability of learning materials among toddlers receiving pediatric primary care in two FQHCs. Examining this population provides an opportunity to describe structural aspects of the home learning environment among families who may experience socioeconomic constraints while also recognizing that caregivers are likely to provide structural resources within their homes for the development of their children. The objectives were to (1) describe caregiver-reported availability of developmentally appropriate learning materials across each domain of the ALM subscale of the StimQ_2_ Toddler and (2) examine within-family changes in reported availability of materials within these domains over a three-month period. Because children’s developmental abilities, interests, and play needs evolve rapidly during toddlerhood (14, 29), we expected caregiver-reported availability of learning materials to change over the study period, though the direction and magnitude of such change was not hypothesized as this study was intended to be descriptive in nature.

## Methods

### Study design

This study was a secondary analysis from a larger pilot study designed to evaluate recruitment and retention strategies for Prescription for Play (P4P), a play promotion intervention delivered during routine 18–36-month well-child checks (WCCs). The P4P program has been described in detail elsewhere (22, 23). Briefly, P4P trains pediatric healthcare providers to counsel caregivers on the importance of play and supplies toy kits and educational materials that providers distribute during WCCs to support continued play after the visit. The intervention is brief and delivered during a single session in conjunction with standard anticipatory guidance during a routine visit. Although P4P promotes caregiver-child play and provides families with play-related resources, the intervention was not specifically designed to increase the availability of home learning materials.

The parent study included caregivers who either received or did not receive the P4P intervention during their child’s 18–36-month WCC. To support implementation in real-world settings, participants were not randomized. Instead, the participating FQHCs assigned clinics within their organization to serve as intervention or control sites, such that families receiving care at intervention sites were administered P4P and those receiving care at control sites were not.

### Setting

Caregivers were recruited from two FQHCs located in Florida and New York that primarily serve families experiencing socioeconomic disadvantage. The participating health centers served populations with substantial socioeconomic needs (24). In Florida, 68.5% of the patient population was uninsured or enrolled in Medicaid, and 65.3% were at or below 100% of the federal poverty level. In New York, 75.8% of the patient population was uninsured or enrolled in Medicaid, and 82.4% were at or below 100% of the federal poverty level.

### Participants and procedures

Caregivers of children aged 18–36 months attending routine WCCs at two FQHCs in Florida and New York were recruited. Eligible caregivers were at least 18 years of age, identified as the child’s primary caregiver, and had access to either a telephone or email address to complete study surveys. Caregivers who were unable to complete the survey in English, Spanish, or Haitian Creole or who lacked telephone or email access were not eligible to participate. During their child’s routine WCC, pediatric healthcare providers introduced the study using a standardized recruitment script and provided caregivers with an information sheet describing the study purpose, procedures, risks, benefits, and multiple methods for opting out of future contact. Providers at intervention sites then delivered the P4P intervention while those at the control sites conducted the visit as usual.

Caregivers who did not opt out had their contact information securely transferred to an independent survey vendor (Crossroads Group, Inc.), which managed participant contact and survey administration. The survey vendor contacted eligible caregivers by telephone or email within 1 month of the WCC. Surveys were administered in English, Spanish, or Haitian Creole according to caregiver preference. Verbal informed consent was obtained before survey administration. Caregivers who completed the baseline survey were re-contacted approximately 3 months later to complete a follow-up survey using the same procedures. Participants received gift cards for completing each survey. Data were collected between May and December 2024. The study protocol was approved by the Community Health Center, Inc. Institutional Review Board.

### Measures

#### Caregiver characteristics

Caregivers self-reported demographic characteristics through the study questionnaire administered by the survey vendor. Characteristics included age, gender, race/ethnicity, and relationship to the pediatric patient. The language in which the survey was completed was recorded by the survey administrator.

### StimQ_2_ toddler: availability of learning materials

The home learning environment was assessed using the ALM subscale from the validated StimQ_2_ Toddler measure (3, 8). The ALM subscale inventories developmentally appropriate toys and learning materials across five domains: Symbolic Play (10 items; e.g., puppets, costumes), Art (6 items; e.g., crayons, coloring books), Adaptive/Fine Motor (12 items; e.g., shape-sorters, stacking toys), Language (4 items; e.g., toy letters and numbers), and Life-Size Play (5 items; e.g., child-sized table and chair, tricycle). Caregivers indicated whether each item is present in the home. Endorsed items were summed within each domain and converted to categorical scores according to standard StimQ_2_ Toddler scoring procedures (3). Symbolic Play and Adaptive/Fine Motor domains were scored from 0 to 2 points, whereas Art, Language, and Life-Size Play domains were scored from 0 to 1 point. Domain scores were summed to yield a total ALM score ranging from 0 to 7, with higher scores indicating greater reported availability of developmentally appropriate learning materials.

#### Statistical analysis

Analyses were restricted to caregivers who completed both the baseline and three-month follow-up surveys. Missing data were handled using complete-case analysis, such that only caregivers with available data at both time points were included in paired-samples analyses. No imputation methods were applied. To assess potential attrition bias, baseline characteristics and ALM scores of caregivers retained at follow-up were compared with those of caregivers lost to follow-up using independent-samples *t* tests for continuous variables and Pearson chi-square tests or Fisher’s exact tests for categorical variables, as appropriate. These analyses were conducted to describe potential differences between retained caregivers and those lost to follow-up and were not intended to identify predictors of retention or generate adjustment variables.

Because P4P was not designed to intervene on home learning materials, analyses were conducted to determine whether participating caregivers could be reasonably pooled across intervention groups for this descriptive analysis. Baseline differences in caregiver characteristics and ALM scores were first compared between groups, with standardized mean differences (SMDs) calculated using Cohen’s *d* based on pooled standard deviations to describe the magnitude of these differences independent of sample size. Of note, any baseline differences between groups reflect naturally occurring differences that likely resulted from a lack of randomization rather than intervention effects. To account for multiple comparisons across baseline characteristics, Bonferroni-adjusted significance thresholds were applied, with a family-wise error rate of *α* = 0.05. An additional sensitivity analysis was conducted using repeated-measures analysis of variance (ANOVA) to examine whether changes in ALM from baseline to follow-up differed by P4P exposure. Time (baseline vs. follow-up) was included as the within-subject factor and P4P exposure (intervention vs. control group) as the between-subject factor. This analysis was conducted to determine whether observed changes in ALM scores were consistent across intervention and control groups.

After confirming the appropriateness of pooling the intervention and control groups, descriptive statistics were used to summarize caregiver characteristics and ALM scores at baseline and follow-up. The normality of change scores (follow-up minus baseline) was assessed using skewness and kurtosis statistics. All change scores were within the acceptable range (±2.0), supporting the use of paired-samples *t* tests (25). Changes in ALM scores between baseline and follow-up were then examined using paired-samples *t* tests, with mean differences and 95% confidence intervals (CIs) reported. In addition, Cohen’s *d* effect sizes with 95% CIs were calculated to determine the magnitude of change; the proportion of caregivers whose scores increased, remained unchanged, or decreased over time was also calculated for each ALM domain. All statistical analyses were conducted using IBM SPSS Statistics Version 29. Statistical significance was defined as *p* < 0.05. Bonferroni corrections were applied to analyses involving multiple comparisons, as indicated in the corresponding tables.

## Results

### Sample characteristics

A total of 917 caregivers were contacted to participate in the study, of whom 240 (26.2%) completed the baseline survey, and 148 (61.7%) completed the 3-month follow-up. Baseline characteristics of caregivers retained at follow-up (*n* = 148) and those lost to follow-up (*n* = 92) are presented in Supplementary Table S1. Caregivers retained at follow-up did not differ significantly from those lost to follow-up with respect to caregiver age, relationship to child, household type, caregiver gender, race/ethnicity, survey language, or baseline ALM (all *p*s > 0.05). However, retention differed by study site, with greater loss to follow-up at the Florida site compared with New York [*χ*^2^(1) = 10.35, *p* = 0.001]. This difference remained statistically significant after applying a Bonferroni correction (family-wise error rate *α* = 0.05; adjusted *α* = 0.004). Loss to follow-up rates were 51.7% at the Florida site and 30.7% at the New York site.

Of the 148 caregivers who completed both surveys, 38 (25.7%) received the P4P intervention, whereas 110 (74.3%) did not. Baseline demographic characteristics were generally comparable between groups; however, caregivers receiving P4P differed from those not receiving P4P in caregiver race/ethnicity (*p* = 0.01; SMD = 0.65). No significant differences were observed for relationship to child, household type, caregiver gender, age, caregiver language, or study site (Supplementary Table S2). Baseline ALM scores differed between caregivers who received P4P and those who did not, with higher baseline total ALM scores among caregivers receiving P4P (mean = 5.82, SD = 1.39) compared with those who did not receive P4P (mean = 5.06, SD = 1.71; *p* = 0.02; SMD = 0.46). Among individual ALM domains, Adaptive/Fine Motor scores were higher in the P4P group (mean = 1.74, SD = 0.50 vs. 1.41, SD = 0.65; *p* = 0.002; SMD = 0.53), while differences in Symbolic Play, Art, Language, and Life-Size Play were not statistically significant (Supplementary Table S2). After applying a Bonferroni correction (family-wise error rate *α* = 0.05; adjusted *α* = 0.004), only the baseline difference in Adaptive/Fine Motor scores remained statistically significant.

Repeated-measures ANOVA models examining changes in ALM over time demonstrated no significant Time × Group interactions for total ALM or any individual ALM domains (all *p*s >0.05; Supplementary Table S3). These results indicate that changes in ALM over time were similar among caregivers who did and did not receive P4P, thereby justifying the pooling of participants between groups for this secondary analysis. Sample characteristics for the final pooled sample are reported in Table 1.

**Table 1:** Sample characteristics (*N* = 148).

Characteristic	Total (*N* = 148)
	Mean (SD) or *n* (%)
Relationship to child	
Parent	147 (99.3%)
Grandparent	1 (0.7%)
Household type	
Single parent household	35 (23.6%)
Dual parent household	113 (76.4%)
Caregiver gender	
Women	143 (96.6%)
Men	5 (3.4%)
Caregiver age	31.55 (6.35)
Caregiver race	
Black or African American	14 (9.5%)
White	1 (0.7%)
Hispanic/Latine	130 (87.8%)
Other*	3 (2.0%)
Caregiver language	
English	18 (12.2%)
Spanish	120 (81.1%)
Haitian Creole	10 (6.8%)
Site	
Florida	42 (28.4%)
New York	106 (71.6%)
Participation in prescription for play (P4P)	
No P4P	110 (74.3%)
P4P	38 (25.7%)

*Other includes American Indian or Alaska Native and Asian or Asian American.

### Baseline ALM characteristics and availability of learning materials

Caregivers reported moderate-to-high availability of learning materials across ALM domains (Table 2A). Moderate-to-high availability was defined according to the standard StimQ_2_ Toddler ALM scoring procedures (Symbolic Play and Adaptive/Fine Motor: ≥5 items; Art, Language, and Life-Size Play: ≥2 items). At baseline, the proportion of caregivers meeting the threshold for moderate-to-high availability ranged from 56.8% for Adaptive/Fine Motor to 79.7% for Art, with higher proportions observed at follow-up across all five domains, ranging from 63.5% for Adaptive/Fine Motor materials to 91.9% for Art materials. Representative item-level endorsement (Table 2B) shows the most and least commonly reported items within each domain at baseline. Across domains, Art materials and Symbolic Play materials were among the most commonly available resources. At baseline, coloring books (77.0%) and small cars, trucks, or trains (95.9%) were frequently reported, whereas some materials requiring specialized space or equipment were less commonly available. For example, life-size play areas (20.3%) and costumes for dress-up (23.0%) were among the least frequently endorsed items at baseline. Complete item-level endorsement frequencies for all 37 ALM items are provided in Supplementary Table S4.

**Table 2:** Baseline and follow-up availability of learning materials (ALM) by domain and representative item-level endorsement (*N* = 148).

Measure	Baseline *n* (%)	Follow-up *n* (%)
2A. Moderate-to-high availability by ALM subdimension		
Symbolic Play (≥5 items)	95 (64.2)	127 (85.8)
Adaptive/Fine Motor (≥5 items)	84 (56.8)	94 (63.5)
Art (≥2 items)	118 (79.7)	136 (91.9)
Language (≥2 items)	90 (60.8)	102 (68.9)
Life-Size Play (≥2 items)	107 (72.3)	119 (80.4)
2B. Representative item-level endorsement		
Symbolic play		
Most: Small cars, trucks, or trains	142 (95.9)	145 (98.0)
Least: Costumes for dress-up	34 (23.0)	59 (39.9)
Adaptive/fine motor		
Most: Toy musical instrument	107 (72.3)	110 (74.3)
Least: Large plastic beads or links	15 (10.1)	28 (18.9)
Art		
Most: Coloring books	114 (77.0)	131 (88.5)
Least: Colorful clay (e.g., Play-Doh)	41 (27.7)	53 (35.8)
Language		
Most: Toy that says object names, alphabet letters, or animal sounds	92 (62.2)	110 (74.3)
Least: Children’s matching/card game	52 (35.1)	49 (33.1)
Life-size play		
Most: Child-sized table and chair	94 (63.5)	93 (62.8)
Least: Life-size play area (e.g., toy kitchen, workshop)	30 (20.3)	35 (23.6)

Representative items were selected as the single highest- and lowest-endorsed items within each ALM subdimension at baseline, allowing for descriptive comparison at follow-up. Complete item-level endorsement frequencies for all 37 ALM items are provided in Supplementary Table S2.

### Change in availability of learning materials over time

Paired-samples *t*-tests demonstrated an overall increase in caregiver-reported ALM from baseline to 3-month follow-up (Table 3). Total ALM scores increased from a mean of 5.26 (SD = 1.66) at baseline to 5.82 (SD = 1.40) at follow-up, representing a mean increase of 0.57 points (95% CI: 0.36 to 0.78, *p* < 0.001) and a small-to-moderate effect size (Cohen’s *d* = 0.44, 95% CI: 0.27 to 0.61). Changes varied across ALM domains. The largest increase was observed for Symbolic Play, which increased by 0.22 points (95% CI: 0.14 to 0.29, *p* < 0.001; Cohen’s *d* = 0.49, 95% CI: 0.32 to 0.66). Art materials also increased significantly, with a mean increase of 0.12 points (95% CI: 0.05 to 0.19, *p* = 0.007; Cohen’s *d* = 0.28, 95% CI: 0.12 to 0.44). Changes in the remaining domains were smaller and did not reach statistical significance. At the participant level, 65 caregivers (43.9%) demonstrated an increase in total ALM scores, 59 (39.9%) showed no change, and 24 (16.2%) demonstrated a decrease. Domain-level changes were less common, consistent with the restricted scoring range of individual ALM subdomains (0–1 or 0–2 points).

**Table 3:** Changes in availability of learning materials (ALM) subdimensions and overall score from baseline to follow-up (*N* = 148).

Outcome	Score range	Baseline mean (SD)	Follow-up mean (SD)	Mean difference (95% CI)	*p*	Cohen’s *d* (95% CI)	Increased *n* (%)	No change *n* (%)	Decreased *n* (%)
Total ALM	0–7	5.26 (1.66)	5.82 (1.40)	−0.57 (−0.78, −0.36)	<0.001	0.44 (0.27, 0.61)	65 (43.9)	59 (39.9)	24 (16.2)
Symbolic play	0–2	1.64 (0.50)	1.85 (0.38)	−0.22 (−0.29, −0.14)	<0.001	0.49 (0.32, 0.66)	34 (23.0)	112 (75.7)	2 (1.4)
Adaptive/fine motor	0–2	1.49 (0.63)	1.56 (0.63)	−0.07 (−0.17, 0.03)	0.174	0.11 (−0.05, 0.27)	27 (18.2)	106 (71.6)	15 (10.1)
Art	0–1	0.80 (0.40)	0.92 (0.27)	−0.12 (−0.19, −0.05)	0.007	0.28 (0.12, 0.44)	24 (16.2)	118 (79.7)	6 (4.1)
Language	0–1	0.61 (0.49)	0.69 (0.46)	−0.08 (−0.17, 0.01)	0.064	0.15 (−0.01, 0.32)	27 (18.2)	106 (71.6)	15 (10.1)
Life-size play	0–1	0.72 (0.45)	0.80 (0.40)	−0.08 (−0.16, 0.00)	0.051	0.16 (0.00, 0.32)	25 (16.9)	110 (74.3)	13 (8.8)

## Discussion

This study characterized caregiver-reported availability of developmentally appropriate learning materials among toddlers receiving pediatric care in two FQHCs and examined changes in reported availability over a three-month period. Overall, caregivers reported moderate-to-high availability of learning materials across symbolic play, art, adaptive/fine motor, language, and life-size play domains. These findings suggest that many families receiving care in safety-net pediatric settings provide children with a range of material resources that support exploration and early learning despite potential socioeconomic barriers.

The availability of learning materials varied across domains. Symbolic play and art materials were among the most commonly reported resources, whereas life-size play materials and some specialized fine-motor materials were less frequently available. These patterns are consistent with conceptualizations of the home learning environment as a multidimensional construct in which different types of materials provide distinct opportunities for developmental stimulation and may be prioritized differently within homes (1–3). For example, life-size play materials, such as toy kitchens, workshops, and other large play structures, may require greater household space and financial resources, which could partially explain their lower availability.

Importantly, the presence of learning materials should not be interpreted as an absence of socioeconomic challenges or as evidence that structural barriers do not affect children’s developmental opportunities. Prior research has consistently demonstrated that socioeconomic conditions influence children’s access to books, toys, and other learning resources (1, 4, 26). However, socioeconomic disadvantage is not synonymous with a lack of developmental support. Families experiencing economic constraints often demonstrate adaptive caregiving practices, resourcefulness, and strengths that support children’s development (3, 4, 19–21, 27). The present findings align with a strengths-based developmental perspective by documenting existing resources within families while recognizing the broader social and economic conditions that shape access to those resources.

Caregiver-reported availability of learning materials increased modestly over the three-month study period. The largest improvements were observed in total ALM scores, symbolic play materials, and art materials, whereas changes in other domains were smaller and not statistically significant. These findings suggest that structural aspects of the home learning environment may demonstrate short-term variation, although the magnitude of change may differ depending on the type of material assessed. During toddlerhood, children rapidly develop new interests and abilities, and caregivers may introduce or acquire materials that correspond with emerging developmental skills (2, 3, 28). Additionally, families may receive ongoing exposure to pediatric anticipatory guidance that emphasizes play and early learning during routine WCCs, which may influence caregivers’ awareness of developmental resources (13, 18).

Although the overall ALM score increased modestly over time, individual patterns of change varied considerably. Nearly half of caregivers reported increased availability of learning materials, whereas approximately 40% showed no change and a smaller proportion reported decreases. This variability suggests that the availability of learning materials is not uniform across families and may reflect differences in children’s developmental needs, household resources, caregiver priorities, or other contextual factors that were not measured in the present study. These findings underscore the dynamic nature of the home learning environment and highlight the importance of considering individual family contexts when interpreting changes in structural learning resources.

The observed changes should not be interpreted as evidence that the P4P intervention caused increases in learning material availability. The present study was a secondary descriptive analysis and was not designed to evaluate intervention effectiveness. Although some caregivers received P4P, the intervention primarily focused on promoting caregiver–child play rather than specifically increasing the availability of home learning materials. The sensitivity analysis demonstrated that changes in total ALM score did not differ by P4P exposure, further supports interpretation of these findings as descriptive changes over time rather than intervention effects.

### Strengths and limitations

This study contributes to the literature by describing domain-specific patterns of learning material availability among families receiving pediatric care in safety-net settings and by examining within-family changes over a relatively short period. The use of the ALM subscale allowed examination of specific categories of materials rather than relying only on composite measures of cognitive stimulation.

Several limitations should be considered. First, this was a secondary observational analysis of a pilot implementation study, and the descriptive design does not allow causal conclusions regarding changes in learning material availability. Observed changes may reflect normal developmental progression, caregiver acquisition of materials, routine pediatric interactions, participation in the study, or other unmeasured household changes. Second, the sample size was modest, and attrition between baseline and follow-up may have introduced selection bias. Although caregivers retained at follow-up and those lost to follow-up did not differ significantly on measured baseline characteristics or ALM scores, retention differed by study site, suggesting that unmeasured site-specific factors may have influenced participation. Third, the three-month follow-up period limits conclusions regarding longer-term stability or trajectories of change in the home learning environment. Fourth, findings may not generalize to all families experiencing socioeconomic disadvantage or to healthcare settings outside of FQHCs. Although the study was conducted in FQHCs serving predominantly low-income and publicly insured populations, individual-level socioeconomic characteristics were not collected, limiting our ability to further characterize socioeconomic variation within the sample or examine whether socioeconomic factors influenced changes in learning material availability. Therefore, findings are most applicable to families receiving pediatric primary care in FQHC settings serving populations with similar sociodemographic characteristics. Finally, ALM is a caregiver-report measure that assesses the presence of learning materials but does not assess how frequently materials are used, caregiver-child interaction quality, or children’s developmental outcomes. In addition, the categorical nature and limited range of some ALM subscales may reduce sensitivity to small changes over time, and findings related to individual domains should therefore be interpreted with caution. Future studies incorporating observational measures or measures of caregiver engagement are needed to better understand how the availability and use of learning materials contribute to children’s developmental experiences.

### Implications and future directions

These findings have several implications for pediatric practice and family-centered interventions. The results suggest that pediatric providers serving families in safety-net settings can build upon existing caregiver strengths by discussing play, learning materials, and developmental activities during routine WCCs. Rather than assuming limited developmental resources, clinicians may use strengths-based approaches that recognize materials and practices already present in families’ homes while identifying areas where additional support may be beneficial. The domain-specific findings may help guide targeted supports. For example, because adaptive/fine motor and life-size play materials were less consistently available than art and symbolic play materials, programs such as toy-lending libraries, community play programs, or resource navigation efforts may consider prioritizing materials that support motor development, problem solving, and pretend play. However, future research should evaluate whether increasing access to specific materials improves caregiver-child engagement or child developmental outcomes. Additional longitudinal research is needed to examine how availability of learning materials changes across early childhood and how structural resources interact with caregiver behaviors, family routines, and broader social determinants of health. Understanding these relationships may help identify effective approaches for supporting early development while recognizing the strengths and resources already present within families.

## Conclusion

This study provides a strengths-based description of structural aspects of the home learning environment among toddlers receiving care in safety-net pediatric settings. Caregivers reported the availability of diverse learning materials across multiple developmental domains, with modest increases observed over a three-month period. These findings highlight that families experiencing socioeconomic disadvantage may provide meaningful developmental resources for their children while also emphasizing opportunities to address gaps in access to specific types of learning materials. Pediatric settings, including FQHCs, may serve as important points of connection for supporting families through developmentally focused counseling, resource distribution, and family-centered approaches that promote early learning. Although average ALM scores increased over time, caregivers demonstrated substantial individual variability in patterns of change. Future longitudinal studies should examine factors associated with these differences, including household resources, caregiver practices, and other contextual influences, to better understand why some families increase, maintain, or decrease the availability of learning materials over time.

## Data Availability

The raw data supporting the conclusions of this article will be made available by the authors upon reasonable request, without undue reservation.
