# Supplementary material for: Caregiver-reported availability of home learning materials for toddlers in underserved communities
Source: Front Public Health. 2026 Aug 19;14:1899274. doi: 10.3389/fpubh.2026.1899274 (PMC13533981; doi:10.3389/fpubh.2026.1899274)
Supplement: Supplementary file 1 [file Table_1.docx]

**Supplemental Table S1**

**Table S1. Baseline characteristics of caregivers retained at 3-month follow-up and caregivers lost to follow-up.**

| **Characteristic** | **Retained participants (n=148)**  *Mean (SD) or n (%)* | **Lost to follow-up (n=92)**  *Mean (SD) or n (%)* | *p* |
| --- | --- | --- | --- |
| *Relationship to child* |  |  | .28 |
| Parent | 147 (99.3%) | 90 (97.8%) |  |
| Grandparent | 1 (0.7%) |  |  |
| Other |  | 1 (1.1%) |  |
| *Household type* |  |  | .75 |
| Single parent household | 35 (23.6%) | 22 (23.9%) |  |
| Dual parent household | 113 (76.4%) | 63 (68.5%) |  |
| *Caregiver gender* |  |  | .50 |
| Women | 143 (96.6%) | 81 (88%) |  |
| Men | 5 (3.4%) | 5 (5.4%) |  |
| *Caregiver age* | 31.55 (6.35) | 31.77 (5.97) | .80 |
| *Caregiver race* |  |  | .14 |
| Black or African American | 14 (9.5%) | 11 (12%) |  |
| White | 1 (0.7%) | 4 (4.3%) |  |
| Hispanic/Latine | 130 (87.8%) | 67 (72.8%) |  |
| Other^*^ | 3 (2.0%) | 1 (1.1%) |  |
| *Caregiver language* |  |  | .12 |
| English | 18 (12.2%) | 18 (19.6%) |  |
| Spanish | 120 (81.1%) | 64 (69.6%) |  |
| Haitian Creole | 10 (6.8%) | 10 (10.9%) |  |
| Site |  |  | .002 |
| Florida | 42 (28.4%) | 45 (48.9%) |  |
| New York | 106 (71.6%) | 47 (51.1%) |  |
| *Participation in Prescription for Play (P4P)* |  |  | .88 |
| No P4P | 110 (74.3%) | 67 (72.8%) |  |
| P4P | 38 (25.7%) | 25 (27.2%) |  |
| *Baseline Availability of Materials* |  |  |  |
| ALM | 5.26 (1.66) | 5.54 (1.53) | .19 |
| Symbolic Play | 1.64 (0.50) | 1.69 (0.49) | .39 |
| Adaptive/Fine Motor | 1.49 (0.63) | 1.56 (0.62) | .42 |
| Art | 0.80 (0.40) | 0.87 (0.34) | .15 |
| Language | 0.61 (0.49) | 0.64 (0.48) | .65 |
| Life-Size Play | 0.72 (0.45) | 0.78 (0.42) | .32 |
| ^*^Other includes American Indian or Alaska Native and Asian or Asian American.  Note: Continuous variables were compared using independent-samples *t* tests. Categorical variables were compared using Pearson χ² tests or Fisher's exact tests, as appropriate. Percentages are based on non-missing data. To account for multiple comparisons, a Bonferroni correction was applied, resulting in a family-wise error rate of α = .05 and a Bonferroni-adjusted significance threshold of α = .004. Under this criterion, only study site differed significantly between caregivers retained at 3-month follow-up and those lost to follow-up. Percentages are based on non-missing data. Missing baseline data among participants lost to follow-up were as follows: relationship to child (*n* = 1), household type (*n* = 7), caregiver gender (*n* = 6), caregiver race/ethnicity (*n* = 9), and baseline availability of materials (*n* = 1). | | | |

**Supplementary Table S2**

**Table S2. Baseline characteristics of the analytic sample by Prescription for Play**

| **Characteristic** | **Total (N=148)** | **P4P (n=38)** | **No P4P (n=110)** | ***p*** | **SMD** |
| --- | --- | --- | --- | --- | --- |
| *Relationship to child* |  |  |  | 1.00 | 0.11 |
| Parent | 147 (99.3%) | 38 (100.0%) | 109 (99.1%) |  |  |
| Grandparent | 1 (0.7%) | 0 (0.0%) | 1 (0.9%) |  |  |
| *Household type* |  |  |  | 0.38 | 0.17 |
| Single parent household | 35 (23.6%) | 11 (29.0%) | 24 (21.8%) |  |  |
| Dual parent household | 113 (76.4%) | 27 (71.1%) | 86 (78.2%) |  |  |
| *Caregiver gender* |  |  |  | 1.00 | 0.06 |
| Women | 143 (96.6%) | 37 (97.4%) | 106 (96.4%) |  |  |
| Men | 5 (3.4%) | 1 (2.6%) | 4 (3.6%) |  |  |
| *Caregiver age, mean (SD)* | 31.55 (6.35) | 31.97 (5.81) | 31.41 (6.55) | 0.64 | 0.09 |
| *Caregiver race* |  |  |  | 0.01 | 0.65 |
| Black or African American | 14 (9.5%) | 3 (7.9%) | 11 (10.0%) |  |  |
| White | 1 (0.7%) | 1 (2.6%) | 0 (0.0%) |  |  |
| Hispanic/Latine | 130 (87.8%) | 31 (81.6%) | 99 (90.0%) |  |  |
| Other* | 3 (2.0%) | 3 (7.9%) | 0 (0.0%) |  |  |
| *Caregiver language* |  |  |  | 0.07 | 0.43 |
| English | 18 (12.2%) | 7 (18.4%) | 11 (10.0%) |  |  |
| Spanish | 120 (81.1%) | 31 (81.6%) | 89 (80.9%) |  |  |
| Haitian Creole | 10 (6.8%) | 0 (0.0%) | 10 (9.1%) |  |  |
| *Site* |  |  |  | .15 | 0.30 |
| Florida | 42 (28.4%) | 7 (18.4%) | 35 (31.8%) |  |  |
| New York | 106 (71.6%) | 31 (81.6%) | 75 (68.2%) |  |  |
| *Baseline Availability of Materials* |  |  |  |  |  |
| ALM | 5.26 (1.66) | 5.82 (1.39) | 5.06 (1.71) | .02 | 0.46 |
| Symbolic Play | 1.64 (.50) | 1.71 (0.46) | 1.61 (0.51) | .26 | 0.20 |
| Adaptive/Fine Motor | 1.49 (.63) | 1.74 (0.50) | 1.41 (0.65) | .002 | 0.53 |
| Art | .80 (.40) | 0.84 (0.37) | .78 (0.42) | .43 | 0.15 |
| Language | .61 (.49) | 0.76 (0.43) | 0.55 (0.50) | .02 | 0.43 |
| Life-Size Play | .72 (.45) | 0.76 (0.43) | 0.71 (0.46) | .52 | 0.12 |
| *Other includes American Indian or Alaska Native and Asian or Asian American.  **Note:** Continuous variables were compared using independent-samples *t* tests. Categorical variables were compared using Pearson χ² tests or Fisher's exact tests, as appropriate. SMD = standardized mean difference. For continuous variables, SMDs were calculated using Cohen's *d* based on pooled standard deviations. For categorical variables, SMDs were calculated based on differences in category proportions between groups. To account for multiple comparisons, a Bonferroni correction was applied, resulting in a family-wise error rate of α = .05 and a Bonferroni-adjusted significance threshold of α = .004. After correction, only the difference in Adaptive/Fine Motor scores remained statistically significant. | | | | | |

**Supplementary Table S3**

**Table S3. Sensitivity analysis examining changes in Availability of Learning Materials by Prescription for Play (P4P) exposure**

| **Availability of Learning Materials (ALM)** | **P4P** | **No P4P** | **Time × P4P interaction**  ***F*** | ***p*** | **Partial *η²*** |
| --- | --- | --- | --- | --- | --- |
| **Total ALM** |  |  |  |  |  |
| Baseline, mean (SD) | 5.82 (1.39) | 5.06 (1.71) |  |  |  |
| Follow-up, mean (SD) | 6.37 (1.15) | 5.64 (1.44) |  |  |  |
| Mean change | +0.55 | +0.57 | 0.007 | 0.934 | <.001 |
| **Symbolic Play** |  |  |  |  |  |
| Baseline, mean (SD) | 1.71 (0.46) | 1.61 (0.51) |  |  |  |
| Follow-up, mean (SD) | 1.95 (0.23) | 1.82 (0.41) |  |  |  |
| Mean change | +0.24 | +0.21 | 0.109 | 0.741 | .001 |
| **Adaptive/Fine Motor** |  |  |  |  |  |
| Baseline, mean (SD) | 1.74 (0.50) | 1.41 (0.65) |  |  |  |
| Follow-up, mean (SD) | 1.76 (0.54) | 1.49 (0.65) |  |  |  |
| Mean change | +0.02 | +0.08 | 0.239 | 0.626 | .002 |
| **Art** |  |  |  |  |  |
| Baseline, mean (SD) | 0.84 (0.37) | .78 (0.42) |  |  |  |
| Follow-up, mean (SD) | 0.97 (0.16) | 0.90 (0.30) |  |  |  |
| Mean change | +0.13 | +0.12 | 0.027 | 0.871 | <.001 |
| **Language** |  |  |  |  |  |
| Baseline, mean (SD) | 0.76 (0.43) | 0.55 (0.50) |  |  |  |
| Follow-up, mean (SD) | 0.82 (0.39) | 0.65 (0.48) |  |  |  |
| Mean change | +0.06 | +0.10 | 0.147 | .702 | .001 |
| **Life-Size Play** |  |  |  |  |  |
| Baseline, mean (SD) | 0.76 (0.43) | 0.71 (0.46) |  |  |  |
| Follow-up, mean (SD) | 0.87 (0.34) | 0.78 (0.42) |  |  |  |
| Mean change | +0.11 | +0.07 | 0.118 | .732 | .001 |
| **Note.** Results are from a repeated-measures ANOVA with Time (baseline vs. follow-up) as the within-subject factor and Prescription for Play (P4P) exposure (intervention vs. control group) as the between-subject factor. To account for multiple comparisons, a Bonferroni correction was applied, resulting in a family-wise error rate of α = .05 and a Bonferroni-adjusted significance threshold of α = .008. The non-significant Time × P4P interaction indicates that changes in ALM over time did not differ between caregivers who received P4P and those who did not. | | | | | |

**Supplementary Table S4**

**Table S4. Item-level endorsement of Availability of Learning Materials at baseline and follow-up**

| **Item** | **Baseline *n*** | **Follow-up *n*** | **Change** |
| --- | --- | --- | --- |
| **Symbolic Play Materials** | | | |
| Doll or action figure | 120 | 123 | +3 |
| Puppet, hand puppet, or marionette | 42 | 41 | −1 |
| Costume for child to dress up in | 34 | 59 | +25 |
| Small housekeeping toy (e.g., broom, vacuum, lawn mower) | 68 | 88 | +20 |
| Toy pots, pans, or dishes or tea set (must be child’s toy, not discarded kitchen utensils) | 75 | 86 | +11 |
| Small car, truck, or train | 142 | 145 | +3 |
| Imaginary play activity set (e.g., Fisher-Price barn, garage, house, doll house) | 60 | 74 | +14 |
| Toy food (e.g., fruits, vegetables, hamburgers, sandwiches, pizza) made of plastic or other toy material | 91 | 111 | +20 |
| Toy school bus or other vehicle with little people that fit into it | 101 | 107 | +6 |
| Set of small toy animals made for young children to play with, not stuffed animals | 109 | 109 | 0 |
| **Art Materials** | | | |
| Crayons | 112 | 134 | +22 |
| Coloring book | 114 | 131 | +17 |
| Chalkboard with chalk or dry erase board with markers that can be erased | 56 | 76 | +20 |
| Colorful clay that can be molded into different shapes (e.g., Play-Doh) | 41 | 53 | +12 |
| Magic marker set or colored pencil set specifically set aside for child to use | 82 | 96 | +14 |
| **Fine Motor Materials** | | | |
| Wood puzzles with 1 to 7 pieces made especially for young children | 60 | 77 | +17 |
| Shape sorter toy container with openings to fit different shaped blocks | 93 | 83 | −10 |
| Colored plastic rings of different sizes that stack on pole | 58 | 75 | +17 |
| Nesting/stacking toy, toys of different sizes that fit into or on top of each other | 75 | 82 | +7 |
| Plastic or wooden beads that child puts string or rope through | 21 | 36 | +15 |
| Set of wooden or plastic blocks made for young child | 91 | 95 | +4 |
| Pop-up toy child presses or moves knob and characters or other items pop up | 38 | 49 | +11 |
| Pail and shovel | 94 | 78 | −16 |
| Large colorful building blocks of varied shapes that fit together to build | 55 | 62 | +7 |
| Toy cash register that has numbers that pop up when pressed or plastic coins | 29 | 36 | +7 |
| Large plastic beads or links that snap together to form a chain and then pull apart | 15 | 28 | +13 |
| Toy musical instrument such as toy xylophone, toy drum, or toy piano keyboard | 107 | 110 | +3 |
| **Language Materials** | | | |
| Set of toy letters, complete alphabet, made of plastic or wood | 69 | 87 | +18 |
| Set of toy numbers, at least 1–9, made of plastic or wood | 77 | 86 | +9 |
| Children’s card game to name or match pictures, shapes, colors, words, letters, or numbers | 52 | 49 | −3 |
| Toy which says name of object or letter of alphabet or makes animal sounds when child pushes button | 92 | 110 | +18 |
| **Life-Size Materials** | | | |
| Tricycle | 71 | 78 | +7 |
| Big toy car or animal that child can ride or rock | 85 | 85 | 0 |
| Life-size toy play area such as toy kitchen, washer/dryer, workshop, tool bench | 30 | 35 | +5 |
| Child-size table and chair | 94 | 93 | −1 |
| Large toy that child can push around or carry things, such as shopping cart | 85 | 91 | +6 |
